# Cortical morphology predicts placebo response in multiple sclerosis

**DOI:** 10.1038/s41598-021-04462-7

**Published:** 2022-01-14

**Authors:** Mariya V. Cherkasova, Jessie F. Fu, Michael Jarrett, Poljanka Johnson, Shawna Abel, Roger Tam, Alexander Rauscher, Vesna Sossi, Shannon Kolind, David K. B. Li, A. Dessa Sadovnick, Lindsay Machan, J. Marc Girard, Francois Emond, Reza Vosoughi, Anthony Traboulsee, A. Jon Stoessl

**Affiliations:** 1 Department of Medicine (Division of Neurology), Djavad Mowafaghian Centre for Brain Health, University of British Columbia, Vancouver, BC Canada; 2 Department of Radiology, University of British Columbia, Vancouver, BC Canada; 3 Department of Physics and Astronomy, University of British Columbia, Vancouver, BC Canada; 4 Depatment of Pediatrics (Division of Neurology), University of British Columbia, Vancouver, BC Canada; 5 Population Data BC, University of British Columbia, Vancouver, BC Canada; 6 School of Biomedical Engineering, University of British Columbia, Vancouver, BC Canada; 7 Department of Medical Genetics, University of British Columbia, Vancouver, BC Canada; 8 Centre Hospitalier de L’Université de Montréal, Montréal, QC Canada; 9 CHU de Québec-Université Laval, Hôpital de L’Enfant-Jésus, Québec, Canada; 10 Department of Internal Medicine (Neurology), University of Manitoba, Winnipeg, Canada; 11 Department of Psychology, University of British Columbia, Vancouver, Canada; 12 Department of Psychology, West Virginia University, 2128 Life Science Building, Morgantown, WV 26506 USA

**Keywords:** Neuroscience, Neurology, Multiple sclerosis

## Abstract

Despite significant insights into the neural mechanisms of acute placebo responses, less is known about longer-term placebo responses, such as those seen in clinical trials, or their interactions with brain disease. We examined brain correlates of placebo responses in a randomized trial of a then controversial and now disproved endovascular treatment for multiple sclerosis. Patients received either balloon or sham extracranial venoplasty and were followed for 48 weeks. Venoplasty had no therapeutic effect, but a subset of both venoplasty- and sham-treated patients reported a transient improvement in health-related quality of life, suggesting a placebo response. Placebo responders did not differ from non-responders in total MRI T2 lesion load, count or location, nor were there differences in normalized brain volume, regional grey or white matter volume or cortical thickness (CT). However, responders had higher lesion activity. Graph theoretical analysis of CT covariance showed that non-responders had a more small-world-like CT architecture. In non-responders, lesion load was inversely associated with CT in somatosensory, motor and association areas, precuneus, and insula, primarily in the right hemisphere. In responders, lesion load was unrelated to CT. The neuropathological process in MS may produce in some a cortical configuration less capable of generating sustained placebo responses.

## Introduction

Current understanding of the neurobiology of placebo effects comes primarily from laboratory studies of acute placebo interventions. Longer term placebo responses, such as those in clinical trials, have been less studied but appear to rely on structural and functional brain connectivity^1–4^ and involve modulation of fMRI-derived^5^ and metabolic networks^6–8^.

Unlike acute laboratory placebo responses, often studied in healthy participants, those of patients with chronic conditions in clinical trials and real-world settings may reflect a yearning for improvement, tempered by varying levels of hope, prior therapeutic experiences and acceptance of risk for a chance at recovery. In neurobehavioural disorders, placebo mechanisms may interact with neuropathological processes. For example, Alzheimer’s patients show a reduced capacity for placebo analgesia, which has been linked to disrupted connectivity of the prefrontal cortex with the rest of the brain^9^. However, little is known about the interactions between placebo responses and other brain diseases.

To address this question, we examined neuropathological and structural neural correlates of placebo responses of multiple sclerosis (MS) patients undergoing a randomized clinical trial (RCT) of a controversial extracranial venoplasty procedure dubbed the “liberation therapy”. The treatment was based on the hypothesis, now considered disproven, that chronic cerebral spinal venous insufficiency (CCSVI) contributes to MS pathogenesis^10^. Owing to the initially promising results of uncontrolled, unblinded studies^11–16^ and the associated publicity, many patients viewed it as a potential cure and sought it out despite the potential risks and the well warranted skepticism of the scientific community. However, venoplasty subsequently proved ineffective in two double-blind sham-controlled RCTs, one by the pioneers of the procedure^17^, and the other by our group^18^. In the latter, while venoplasty was not superior to sham venoplasty on any outcome measure, a subset of both venoplasty- and sham-treated patients experienced a significant transient improvement in self-reported health-related quality of life suggesting a placebo response. This presented a unique opportunity to examine brain correlates of placebo responses in an MS clinical trial.

**Figure 1:**
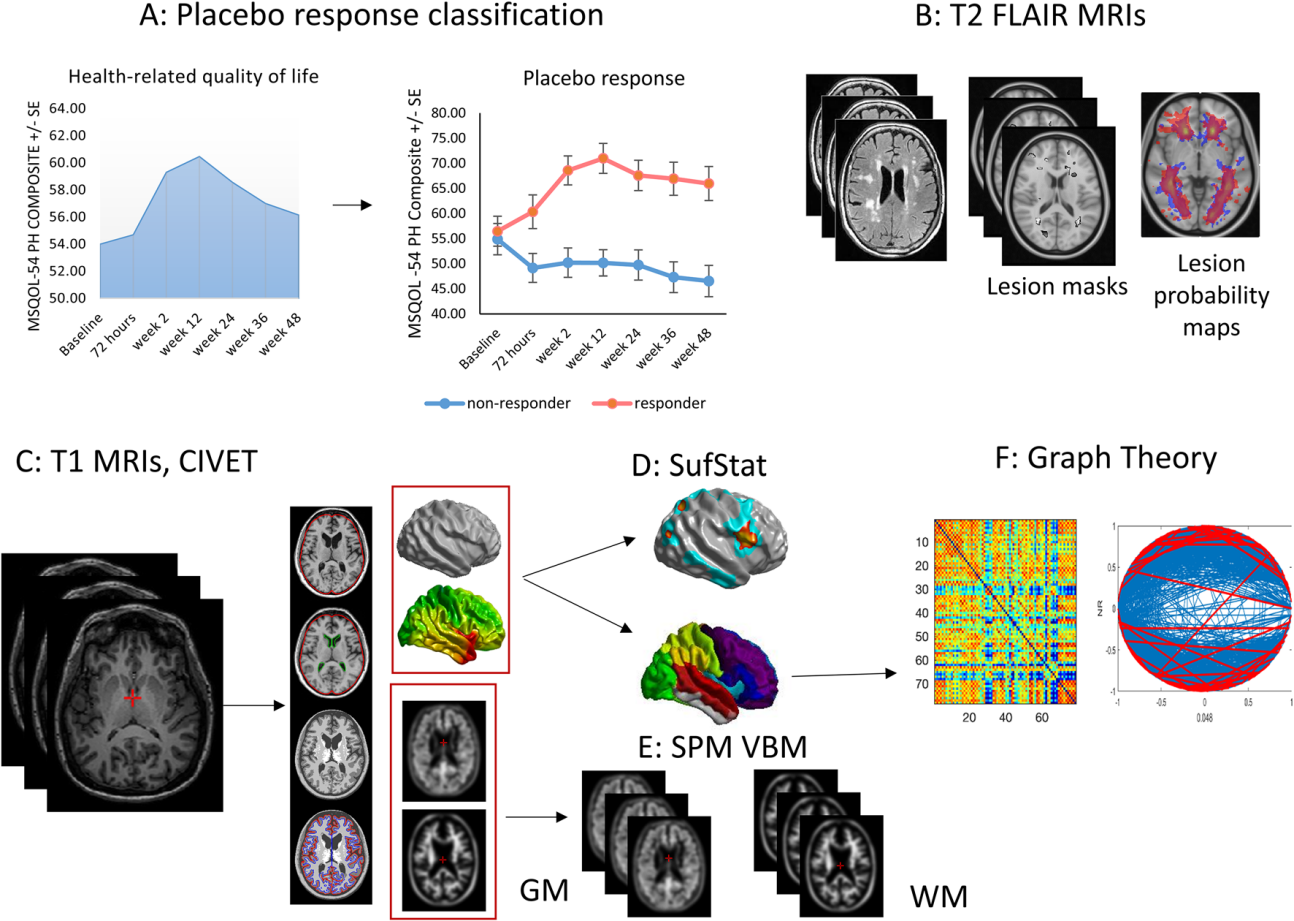
Method overview.

We examined potential MRI-based predictors of this placebo response. Besides standard morphometric analyses (Fig. 1B,C,D,E), we performed a graph theoretical analysis of cortical thickness (CT) covariance (Fig. 1F) to characterize its inter-regional relationships. Graph theory is a modality invariant framework that represents complex systems as networks and describes their organization using a set of common metrics. In graph theoretical terms, the brain is viewed as a network of regions (“nodes”) connected via links (“edges”) representing white matter tracts, structural covariance, or functional connections. While the neurobiological significance of structural covariance networks and more specifically CT networks is not entirely clear, CT is known to covary between structurally and functionally connected regions^19^. This covariation appears to reflect stronger synaptic connectivity between regions that are microstructurally similar^20,21^. Previous studies applying graph theory to CT covariance in MS have found increased network segregation and enhancement of local properties in early disease^22,23^ with a shift in both local and global properties towards more “regular” or uniform networks with advancing disease^24,25^. Studies of diffusion tensor imaging (DTI) based structural networks^26,27^ and functional connectivity^24^ yielded convergent findings. We hypothesized that CT covariance networks would be more anomalous in placebo non-responders, with a shift towards more “regular” graphs with more segregation and less integration. We specifically focused on three key graph metrics of network segregation and integration: clustering coefficient—a measure of segregation; pathlength—a measure of integration; and the small-world index—a derivative measure describing overall network topology.

## Methods

### Participants

Participants with relapsing remitting (RRMS), secondary (SPMS) and primary progressive (PPMS) MS were recruited between May 29, 2013 and Aug 19, 2015 from four Canadian academic centers. Inclusion criteria were: age 18–65 years, diagnosis of definite MS by the 2010 McDonald criteria^28^, an Expanded Disability Status Score (EDSS)^29^ between 0 (i.e. minimal disability) and 6.5 (i.e. using bilateral aids to walk), neurologically stable disease within the 30 days prior to screening, and fulfillment of at least two ultrasound criteria for CCSVI. See^18^ for a detailed description of the trial’s methods and entry criteria. Participants on standard disease-modifying therapies were permitted to continue on the medication, and changes were allowed for on study relapses after randomization. The data analysis reported in the current manuscript was approved as a sub-study by the Clinical Research Ethics Board of the University of British Columbia. Of the total 104 MS participants, we analyzed the data of 88 who had T1-weighted MRIs of sufficient quality for CT analyses. For the remaining scans, signal intensity at the lateral extremes was too low for successful surface extraction. Of the excluded participants, 8 received the active treatment, the remaining 8 received sham, 12 were female, 11 had RRMS (4 had SPMS, 1 had progressive relapsing MS); average disease duration for he excluded patients was 9.47 (± 5.53) years.

Although the primary purpose of this study was to compare the placebo responders to non-responders in the trial, we used MRIs from 43 sex and age-matched healthy controls (30 females, age: 52.98 ± 8.93) to provide a benchmark for graph theory analysis of placebo responders vs. non-responders to help determine which CT pattern was more normative. Six of these scans were acquired at Site 1; the rest were obtained from the Open Access Series of Imaging Studies repository (OASIS-3, www.oasis-brains.org, RRID:SCR_007385).

### Experimental design and procedure

Eligible participants were randomized 1:1 to either sham or active balloon venoplasty of all narrowed veins under study in a crossover design. Participants were blinded to treatment allocation. Please see^18^ and the Supplement for a detailed description of the surgical procedures.

After randomization and intervention, participants were followed for 48 weeks until crossover with MRI, ultrasound, clinical assessments, and patient-reported outcome scales including Multiple Sclerosis Quality of Life-54 (MSQOL-54)^30^. For MRI, T1 weighted images with and without gadolinium enhancement, T2 weighted images, and fluid attenuated inversion recovery (FLAIR) images were obtained. An MRI protocol was developed for each site in close collaboration with the image analysis core lab in order to maximize image quality and consistency across sites, using criteria such as grey matter/white matter contrast and signal to noise ratio. MRI acquisition parameters are given in Table 1.

**Table 1:** MRI acquisition parameters.

Site	Scanner and scan	Resolution	Voxel size	TR	TE	TI
Site 1	Philips Intera 3T					
	3D T1 weighted	320 × 320 × 200	0.8 × 0.8 × 0.8	6.2	3.0	
	3D T2 weighted	320 × 320 × 200	0.8 × 0.8 × 0.8	2500	363	
	FLAIR	320 × 320 × 200	0.8 × 0.8 × 0.8	8000	337	2400
Site 2	Siemens Verio 3T					
	3D T1 weighted	320 × 320 × 176	0.78 × 0.78 × 0.78	1900	3.4	900
	3D T2 weighted	512 × 512 × 160	0.488 × 0.488 × 1.0	3200	409	
	FLAIR	320 × 320 × 160	0.83 × 0.83 × 0.83	8000	337	2400
Site 3	Philips Achieva 3T					
	3D T1 weighted	320 × 320 × 200	0.8 × 0.8 × 0.8	6.2	3.0	
	3D T2 weighted	320 × 320 × 200	0.8 × 0.8 × 0.8	2500	363	
	FLAIR	320 × 320 × 200	0.8 × 0.8 × 0.8	8000	337	2400
Site 4	Philips Achieva 3T					
	3D T1 weighted	320 × 320 × 112	0.8 × 0.8 × 1.6	6.5	3.2	
	3D T2 weighted	320 × 320 × 112	0.8 × 0.8 × 1.6	2500	255	
	FLAIR	336 × 336 × 112	0.76 × 0.76 × 1.6	4800	330	1650
Controls: Site1	Philips 3T Achieva					
	3D T1 weighted	256 × 250 × 165	1.0 × 1.0 × 1.0	8.1	3.5	
Controls: OASIS	(Siemens TIM Trio 3T)					
	3D T1 weighted	256 × 256 × 176	1.0 × 1.0 × 1.0	2400	3.16	

3 T = 3 Tesla; 3D = 3-dimensional, TR = repetition time; TE = echo time; TI = inversion time; FLAIR = Fluid Attenuated Inversion Recovery; Site 1 control scans were acquired with parallel imaging, SENSE factor 1.5.

### Analyses

The analyses of the morphological predictors of placebo response focused on MRI measures obtained at baseline, directly prior to extracranial venoplasty/sham. Unless otherwise specified, all statistical analyses of morphometric and lesion data included age, sex, age by sex interaction, disease duration, and site as covariates.

#### Placebo response

Change in MSQOL-54 Physical Health Composite was used as the measure of placebo response, as this measure showed a transient significant improvement in the trial^18^. Scores at baseline and the subsequent 6 assessment points were used to compute the area under the curve (AUC) measure for each participant using the Matlab function *trapz* (Fig. 1A). These values were then adjusted for the baseline score using linear regression, as higher baseline scores predicted less change (β =  − 0.91, SE = 0.32, t =  − 2.88, 95% CI =  − 1.53 to  − 0.28, *p* = 0.005), and standardized residuals were used to subdivide participants into placebo responders (scores > 0, M = 0.83, SD = 0.56) and non-responders (scores ≤ 0, M =  − 0.79, SD = 0.56).

#### Lesion analyses

These analyses aimed to determine whether placebo responders differed from non-responders in terms of lesion burden, type, and location (Fig. 1B).

Lesion segmentation was performed on FLAIR images by a trained radiologist blinded to treatment assignment using manual seed point and connected component analysis^31^; lesion activity analysis was carried out by the same radiologist. Lesion load (LL) was calculated as the sum of volumes of all FLAIR lesion masks (mm^3^) for a given patient. Gadolinium enhanced lesions indicating blood brain barrier disruption were identified on T1 gadolinium scans. Newly enhancing lesions were counted as lesions that were enhanced on the current scan but were not enhancing in previous scans; new and newly enlarging lesions on the FLAIR scans were counted as lesions that were respectively absent or stable and smaller in volume in previous assessment points; unique newly active lesions were newly enhancing lesions and non-enhancing new or newly enlarging lesions on a current scan without double counting. Lesion load and its change over the trial’s duration were compared between placebo responders and non-responders with a linear mixed-effects model with our standard covariates using the lme4 R package (RRID:SCR_015654)^32^. Group differences in lesion counts at each assessment point were analyzed with Poisson regressions, as was change in lesion counts over time as a function of group.

The analysis of lesion location was performed in FSL (Functional MRI of the Brain Software Library, http://www.fmrib.ox.ac.uk/fsl, RRID:SCR_002823). Voxel-wise maps representing the probability of each voxel being lesional were compared statistically between the two groups using the *Randomise* algorithm^33^, which uses non-parametric permutation inference to threshold a voxel-wise statistical map produced, in this case, by voxel-wise unpaired t-tests on the two groups (see Supplement for a detailed description of this analysis).

#### MRI processing for brain morphometric and cortical thickness analyses

Patients’ normalized brain volumes (parenchymal volume normalized by intracranial volume) were computed on T1 MRIs using a segmentation-based approach^34^ and compared between placebo responders and non-responders using linear regression with our standard covariates. Percentage brain volume change was computed using an automated in-house method based on parenchymal edge displacement between scans^35^; change over time was compared between responders and non-responders using a linear mixed-effects model (lme4 R package).

Prior to further morphometric analyses, white matter lesions on the patients’ scans were filled with intensities of neighboring white matter voxels using the *Lesion Filling* function in FSL^36^. This reduces intensity contrast within lesion areas and can improve registration and segmentation of MS brains and resulting morphometric measurements^37^. Native T1 MRIs were processed through the CIVET pipeline (version 2.1, Fig. 1C)^38^ housed on the CBRAIN web-based image analysis platform (McGill Centre for Integrative Neuroscience, RRID:SCR_005513^39^). Please see the Supplement for a detailed description of steps in the pipeline. For each participant, the pipeline yielded (1) 8-mm smoothed grey and white matter volumes for subsequent voxel-based morphometry (see Supplement) and (2) cortical surfaces with measures of cortical thickness. Cortical surfaces were extracted as triangulated meshes using the Constrained Laplacian Anatomic Segmentation Using Proximities^40,41^, and CT was measured across 40,962 triangle vertices in each hemisphere^42^. CT maps were then blurred using a 20 mm surface based kernel^43^.

#### Cortical thickness analysis

CT was analyzed statistically using SurfStat (http://www.math.mcgill.ca/keith/surfstat/, RRID:SCR_007081), a Matlab toolbox for the statistical analysis of surface data with linear mixed effects models and random field theory to correct for multiple comparisons in determining vertex and cluster significance (Fig. 1D)^44^. The analyses were performed using Matlab17a. We first modelled vertex-wise CT as a function of group membership (placebo responder vs. non-responder), adjusting for our standard covariates as well as total brain volume and lesion load at baseline:

$$\begin{aligned} CT & = \beta 1 + \beta 2 \, Volume + \beta 3 \, Age + \beta 4Sex + \beta 5Age*Sex \\ & \quad + \beta 6Disease\_Duration + \beta 7 \, Site + \beta 8 \, LL + \beta 9 \, Group \\ \end{aligned}$$

In light of the differential associations of lesion load with cortical thickness in placebo non-responders and responders (see “Results” section), which may be viewed as violating the assumption of homogeneity of regression slopes^45^, the analysis was performed using models with and without lesion load as a covariate, as well as those substituting baseline EDSS as a measure of disease burden. As this did not change the results, we report the findings from the models with lesion load included as a covariate.

We additionally considered whether placebo response as a continuous variable was associated with CT. We tested this by modelling CT as a function of placebo response (AUC measure of change in MSQOL-54), our standard covariates, total brain volume and lesion load at baseline, and baseline MSQOL-54 scores:

$$\begin{aligned} CT & = \beta 1 + \beta 2 \, Volume + \beta 3 \, Age + \beta 4 \, Sex + \beta 5 \, Age*Sex \\ & \quad + \beta 6Disease\_Duration + \beta 7 \, Site + \beta 8 \, LL + \beta 9 \, Baseline \\ & \quad + \beta 10 \, Response \\ \end{aligned}$$

Given our finding of more regular CT graphs in placebo non-responders (see “Results” section), which could arise from more coordinated tissue loss in anatomically connected regions owing to white matter lesions^25^, we explored the associations between CT and lesion load in placebo responders versus non-responders. We first tested the significance of the interaction between lesion load and group.

$$\begin{aligned} CT & = \beta 1 + \beta 2 \, Volume + \beta 3 \, Age + \beta 4 \, Sex + \beta 5 \, Age*Sex \\ & \quad + \beta 6 \, Disease\_Duration \, + \, \beta 7Site \, + \, \beta 8 \, Responder*LL \\ \end{aligned}$$

Once the significance of this interaction was established, we analyzed the associations between CT and lesion load separately in each group.

$$\begin{aligned} CT & = \beta 1 + \beta 2 \, Volume + \beta 3 \, Age + \beta 4 \, Sex + \beta 5 \, Age*Sex \\ & \quad + \beta 6 \, Disease\_Duration + \beta 7Site + \beta 8 \, LL \\ \end{aligned}$$

#### Graph theoretical cortical thickness analysis

Cortical surfaces were parcellated into 78 regions based on Automated Anatomical Labeling (AAL)^46^; subcortical labels were excluded. Mean CT values for each participant were extracted for each of the 78 regions. A linear regression was performed to adjust these CT values for total brain volume, age, sex and the interaction of age and sex for all participants, as well as disease duration, site and lesion load for the MS participants. Again, given differential associations of lesion load with cortical thickness in placebo non-responders and responders, the graph theoretical analyses were performed on the data both with and without lesion load covaried out, as well as on data adjusting for baseline EDSS in place of lesion load as a measure of disease burden. As this did not change the results, we report the results adjusted for lesion load. The resulting residuals were substituted for the raw cortical thickness values to construct inter-regional correlation matrices for placebo responders, non-responders and controls: Rij (i, j = 1, 2 … n, where n is the number of regions). These correlation matrices were then used to construct group-wise binarized networks and compute graph metrics at 30 linearly spaced sparsity thresholds ranging from 0.1 to 0.5. This thresholding approach normalizes each group-level graph to have the same number of edges. Using the Brain Connectivity Toolbox (RRID:SCR_004841)^47^ in Matlab2017a, we computed the following graph metrics: the clustering coefficient and its normalized version, the characteristic pathlength and its normalized version and the small-world index (Table 2).

**Table 2:** Graph theory metrics.

Term	Description	Computation
Clustering coefficient	A fraction of a node’s neighbors that are also neighbours of each other; a measure of clustered connectivity around individual nodes	$C = \frac{1}{n}\sum\nolimits_{i \in n} {C_{i} } = \frac{1}{n}\sum\nolimits_{i \in n} {\frac{{2t_{i} }}{{k_{i} (k_{i} - 1)}}}$
	In the context of CT networks, it reflects uniformity of CT with respect to individual nodes	*n* = the total number of nodes
		*C*_*i*_ = the clustering coefficient of node *i*
		*ki* = the degree of node *i*
		*C*_*i*_ = *0 for k*_*i*_ < 2
Normalized clustering coefficient	Ratio of the mean clustering coefficient *C* and normalization factor *C*_*rnad*_ computed as the mean clustering coefficient of 10 random networks (see below) with the same number of nodes and edges as the tested input network	$C_{norm} = \frac{C}{{C_{rand} }}$
Characteristic pathlength	A measure of network integration representing the number of edges typically required to connect pairs of nodes in the network	$L = \frac{1}{n}\sum\nolimits_{i \in N} {L_{i} } = \frac{1}{n}\sum\nolimits_{i \in N} {\frac{{\mathop \sum \nolimits_{j \in N \cdot j \ne i} d_{ij}^{ - 1} }}{n - 1}}$
	In the context of CT networks, path length represents the number of required indirect correlations surpassing the sparsity threshold	Li = the average distance between node *i* and all other nodes
		*d*_*ij*_ = the distance from node *i* to node *j*
Normalized pathlength	The ratio of characteristic path length *L* and a normalization factor *L*_*rnad*_ based on 10 random networks, as described above	$L_{norm} = \frac{L}{{L_{rand} }}$
Small-world index	Describes a topology featuring numerous short-range connections with an admixture of few long-range connections; balances specialized and distributed processing while minimizing wiring costs. Small-world networks lie on a continuum between *regular* networks, in which each node has the same number of edges, and *random* networks, in which nodes are connected to other nodes with a random probability	$S = \frac{{C_{norm} }}{{L_{norm} }}$

**Figure 2:**
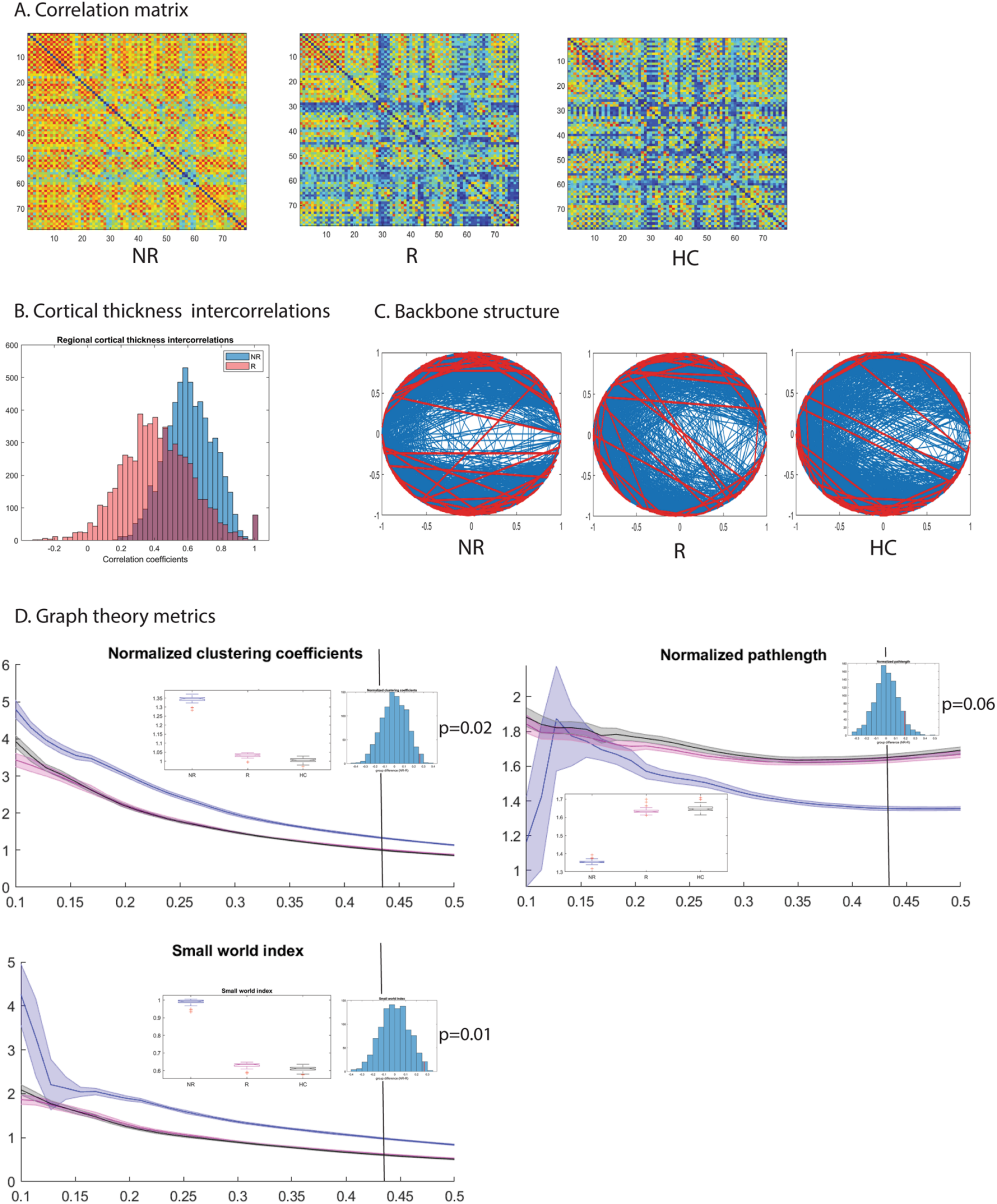
Cortical thickness covariance patterns in placebo responders and non-responders: graph theoretical analysis. (**A**) Correlation matrices of cortical thickness values across 78 cortical areas delineated using Automated Anatomical Labeling (AAL) in placebo non-responders (NR), placebo responders (R), and a group of healthy age- and sex-matched controls (HC). (**B**) A histogram depicting distributions of correlation coefficients in placebo responders and non-responders. (**C**) Backbone structure for correlation matrices in A at the sparsity threshold of 0.43. (**D**) Graph theoretical characteristics of these matrices across the range of sparsity thresholds from 0.1 to 0.5. Error ribbons represent standard deviation for parameter estimates from leave-one-out cross-validation. Box plots represent group comparisons at the sparsity threshold of 0.43 based on leave-one-out cross-validations; histograms represent the p-values based on permutation tests at the sparsity threshold of 0.43.

Leave-one-out cross-validation was performed to estimate the stability of the graph metrics, and error estimates from these cross validations were used to visualize group differences (Fig. 2). Statistical significance of these group differences was evaluated against null distributions of group differences on the metrics based on 1000 random permutations of participants in the input matrix. A Bonferroni correction for multiple comparisons was applied: considering three graph metrics (clustering coefficient, pathlength and small-world index) the α-level was determined to be 0.016. Because normalized and non-normalized variants of clustering coefficient and pathlength are redundant measures, these were not considered as separate comparisons.

### Ethics approval

The clinical research ethics boards at the four participating centers approved the study protocol.

### Patient consent statement

Patients gave written informed consent.

### Consent for publication

Consent to publish was part of informed consent participants provided prior to participation.

## Results

**Figure 3:**
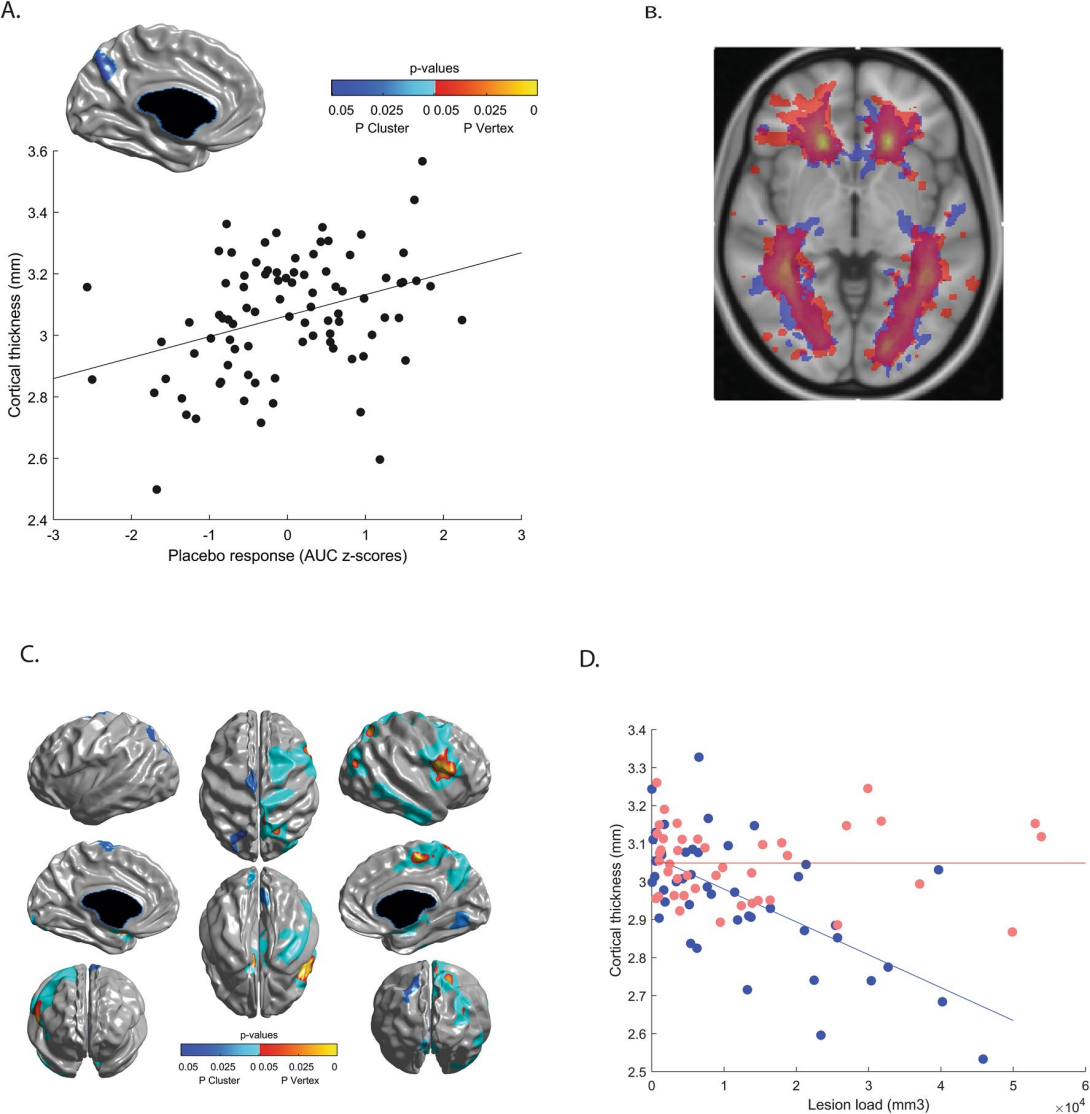
Cortical thickness, white matter lesions and placebo response. (**A**) Precuneus region whose thickness dimensionally predicts a stronger placebo response as depicted in the scatterplot. (**B**) Lesion probability maps in placebo responders (red) and non-responders (blue): no significant differences in lesion location between groups. (**C**) Cortical areas whose thickness was significantly associated with lesion load in the placebo non-responders. (**D**) Mean cortical thickness of the regions shown in relation to white matter lesion load (FLAIR) in the placebo responders (red) and non-responders (blue). A mask of the regions shown in C was used to extract mean CT values across these regions for all participants.

Placebo responders and non-responders did not differ significantly on any demographic or clinical characteristics (Table 3). As expected, the two groups had markedly different subjective treatment response (Fig. 1A). The proportion of placebo responders vs. non-responders did not differ as a function of recruitment site. Placebo responders and non-responders did not differ in terms of normalized brain volumes, regional grey or white matter volume or regional CT. However, there was a significant dimensional association between the magnitude of placebo response and CT of a left precuneus region (x =  − 3.16, y =  − 70.12, z = 37.10; Fig. 3A).

**Table 3:** Demographic and clinical characteristics of placebo responders and non-responders.

	Non-responder (n = 45)	Responder (n = 43)	*p*
Venoplasty, n	24	18	0.19
Sham, n	21	25	
Males, n	18	13	0.24
Females, n	27	30	
Age, mean (SD)	55.0 (7.4)	53.0 (8.5)	0.26
Disease duration, mean (SD)	18.8 (9.6)	16.7 (8.1)	0.29
**MS type (n)**			
RRMS	25	30	0.35
PPMS	6	3	
SPMS	14	10	
Baseline EDSS, median (range)	4.0 (0–6.5)	4.0 (0–6.5)	0.09
Baseline MSFC, mean (SD)	7.2 (6.2)	8.9 (4.9)	0.17
25 foot walk, mean (SD)	15.80 (12.15)	11.87 (7.24)	0.07
9 hole peg test, mean (SD)	108.50 (49.73)	94.41 (36.04)	0.13
PASAT, mean (SD)	37.53 (14.70)	38.63 (14.97)	0.73
Baseline MSQOL-54 PH	55.54 (21.57)	56.96 (18.79)	0.74

RRMS = Remitting relapsing MS; PPMS = primary progressive MS; SPMS = secondary progressive MS; EDSS = expanded disability status scale; MSFC = multiple sclerosis functional composite; PASAT = paced auditory serial addition test; MSQOL-54 PH = multiple sclerosis quality of life-54, physical health composite. For MSFC, raw scores rather than z-scores are given.

### Placebo responders have higher lesion activity

Although most patients did not have gadolinium enhanced lesions, there were significant differences in lesion activity between the groups (Table 4). Placebo responders were more likely to display gadolinium enhanced lesions at baseline (b = 1.26, SE = 0.46, z = 2.73, *p* = 0.006) and to have newly enhancing lesions at 24 weeks (b = 1.26, SE = 0.42, z = 3.01, *p* = 0.003) and at 48 weeks (b = 1.77, SE = 0.51, z = 3.43, *p* = 0.0006). A similar pattern was evident for newly active lesions that were not present on previous gadolinium T1 scans, both at 24 (b = 1.24, SE = 0.27, z = 4.52, *p* < 0.0005) and 48 weeks (b = 1.54, SD = 0.36, z = 4.25, *p* < 0.0005). This included new T2 lesions on FLAIR scans at 24 (b = 1.11, SE = 0.33, z = 3.34, *p* = 0.0008) and 48 weeks (b = 1.57, SE = 0.40, z = 3.97, *p* < 0.0005) and enlarging T2 lesions at 24 weeks (b = 2.61, SE = 0.75, z = 3.49, *p* = 0.0005), though not at 48 weeks (*p* = 0.14).

**Table 4:** Brain morphometric characteristics and lesions.

Measure (SD)	Non-responder (n = 45)			Responder (n = 43)			*p*
New T2 lesions (# of patients with lesion count)	W24		W48	W24		W48	
0	36		36	35		33	< 0.001**
1	4		5	3		4	
2	3		0	1		3	
3	0		2	0		1	
5	1		0	0		0	
6	0		0	1		0	
7	0		0	0		1	
8	0		0	2		0	
9	0		0	1		0	
11	0		0	0		1	

Unique newly active lesions (# of patients with lesion count)	BL	W24	W48	BL	W24	W48	
0	40	33	36	33	34	33	< 0.0005***
1	1	6	5	3	2	3	
2	3	4	0	2	2	3	
3	0	0	1	2	1	1	
4	0	0	0	1	0	0	
5	0	0	1	0	0	0	
6	0	1	0	0	1	1	
7	0	0	0	1	0	0	
8	0	0	0	0	1	1	
11	0	0	0	0	0	1	
15	0	0	0	0	1	0	
22	0	0	0	0	1	0	

Newly enhancing lesions (# of patients with lesion count)	BL	W24	W48	BL	W24	W48	
0	40	36	38	33	36	34	< 0.01*
1	1	6	4	3	4	5	
2	3	2	1	2	0	0	
3	0	0	0	2	1	2	
4	0	0	0	1	0	0	
5	0	0	0	0	0	2	
6	0	0	0	0	1	0	
7	0	0	0	1	0	0	

Newly enlarging T2 lesions (# of patients with lesion count)	W24		W48	W24		W48	
0	42		42	38		38	W24: 0.0005*** W48: 0.14
1	2		0	2		4	
2	0		1	1		1	
7	0		0	1		0	
13	0		0	1		0	

BL = Baseline; W24 = 24 weeks assessment point; W48 = 48 weeks assessment point; WM = white mater. *P* values represent comparisons between placebo responders and non-responders for corresponding assessment points. The models include age, sex, age x sex interaction, disease duration and site as terms.

The group differences were specific to lesion activity. There were no significant differences between placebo responders and non-responders in T2 lesion load either at baseline or at 24-week and 48-week follow-up time points (*p*_s_ ≥ 0.78), and there was no significant increase in lesion load over the 48 weeks (*p* = 0.11). There was no significant change in lesion counts over the trial’s duration, and this did not differ as a function of group (*p*_s_ ≥ 0.2). Finally, there were no significant differences in lesion locations based on a comparison of voxel-wise lesion probability maps (Fig. 3B).

### Placebo non-responders have a more small-world-like CT covariance pattern

Group differences in the metrics presented below were consistent over the range of sparsity thresholds (10–50% sparsity). As input matrices are not stable at low sparsity, which results in high error estimates, we chose a relatively high sparsity threshold of 43% yielding stable input matrices for presenting the results of random permutation tests. *P*-values for the entire range of sparsity thresholds are presented as supporting information (Tables S2–S6); similar *p*-values were seen at most sparsity thresholds.

CT of placebo non-responders was more regionally homogeneous (range r: 0.1–0.95, median r: 0.66) relative to that of responders (range r_s_: − 0.15 to 0.95, median r: 0.49) and controls (range r_s_: − 0.22 to 0.92, median r: 0.41, Fig. 2A,B). Placebo responders did not differ significantly from controls on the computed metrics (*p*_s_ ≥ 0.29). Uncorrected results showed that non-responders had a more segregated network topology with higher mean and normalized clustering coefficients (non-responders vs. responders: *p* = 0.02; non-responders vs. controls: *p* = 0.01). Non-responders also had marginally shorter pathlengths relative to responders (*p* = 0.06) and controls (*p*_s_ < 0.04). This resulted in stronger small-world attributes for non-responders relative to responders and controls (*p*_s_ = 0.01), indicating a shift towards more regular and less random graphs. The group differences in small world index and the difference between non-responders and controls in clustering coefficient survived the correction for multiple comparisons.

### Lesion load is inversely associated with CT only in placebo non-responders

Although lesion load and location did not differ significantly between responders and non-responders, there was a significant difference between the groups in the association between CT and lesion load. While there was no relationship between lesion load and CT in responders, in non-responders, greater lesion load was associated with cortical thinning in 9 clusters (Fig. 3C,D, Table 5). The clusters covered a substantial portion of the right hemisphere including parts of the primary motor and sensory cortices as well as the premotor cortex and somatosensory, visual and auditory association areas. The major clusters included: the primary motor cortex, comprising both the paracentral lobule and the precentral gyrus and extending into the premotor cortex (middle frontal gyrus, Brodmann area BA 6) and the insula; primary somatosensory cortex (postcentral gyrus, BA 3) extending to the superior parietal lobule and the precuneus; superior occipital gyrus (BA 19) extending to middle temporal gyrus (BA 39); middle temporal gyrus (BA 21) extending to inferior temporal gyrus (BA 20). There were also smaller primary motor and superior occipital clusters in the left hemisphere.

**Table 5:** Regions where cortical thickness was predicted by lesion load in placebo non-responders.

Cluster	# Vertices	Peak	x	y	z	t	*p*
Right superior and medial frontoparietal *p* < 0.00005	4080	Medial frontal gyrus (R)	4.30	− 24.62	57.64	5.22	0.008
		Superior parietal lobule (R)	28.69	− 57.71	52.84	5.03	0.013
		Precuneus (R)	13.35	− 66.54	32.83	4.96	0.015
		Middle occipital gyrus (R)	44.45	− 77.33	22.46	4.83	0.021
		Postcentral gyrus (R)	11.82	− 59.08	69.79	4.75	0.027
		Paracentral lobule (R)	13.69	− 41.22	58.15	4.56	0.043
Right precentral gyrus/insula *p* < 0.00005	3026	Precentral gyrus (R)	52.78	− 6.92	10.30	5.32	0.006
		Insula (R)	39.91	− 0.08	16.26	5.21	0.008
Left parahippocampal gyrus *p* < 0.00005	383	Parahippocampal gyrus (L)	− 12.13	− 9.54	− 12.02	5.41	0.005
Right inferior temporal gyrus *p* = 0.0002	1220	Inferior temporal gyrus (R)	59.56	− 7.57	− 28.66	4.51	0.05
Right parahippocampal gyrus *p* = 0.0005	335	Parahippocampal gyrus (R)	15.12	− 12.79	− 11.08	4.82	0.02
Left lingual gyrus *p* = 0.01	246	–	− 16.26	− 93.19	− 12.51	3.14	–
Right lingual gyrus *p* = 0.02	311	–	10.08	− 72.09	− 4.17	4.03	–
Left precentral gyrus *p* = 0.03	385	–	− 14.29	− 19.43	70.91	4.12	–
Left superior parietal lobule *p* = 0.03	329	–	− 31.33	− 70.83	47.50	3.64	–

Significance thresholds for vertices and clusters of contiguous vertices and are determined using Random Field Theory; corrected *p*-values are given. Due to some of the clusters being extensive, the coordinates (in MNI space) are given for either peak vertices for clusters featuring such peaks (labeled in hot colors on Fig. 3). For smaller clusters without peaks, average cluster coordinates are provided.

## Discussion

We examined neural correlates of placebo responses to an investigational procedure, now proven ineffective, which had initially inspired hope in patients, driving many to pursue it despite the potential risks. Our findings revealed significant structural brain differences between those MS patients who experienced a placebo response and those who did not. These findings advance our understanding of both the neurobiology of placebo responses and how the neuropathological changes in MS might impact the propensity to experience them.

While placebo responders and non-responders did not differ significantly in terms of clinical characteristics, lesion load, lesion location, brain volume or regional cortical thickness, the groups differed significantly in terms of their cortical thickness (CT) covariance patterns. Relative to placebo responders, non-responders had more uniform CT across different brain regions with a more clustered topology. Coupled with marginally shorter pathlengths, this resulted in stronger small-world attributes, indicating a shift towards more regular and less random graphs. While a more regular network may be associated with smaller wiring costs, potentially imposed by axonal loss in MS, this type of network may be less capable of distributed processing and functional integration. The absence of differences between responders and controls suggests that the more segregated and regular topology observed in non-responders is anomalous.

Previous studies have suggested that CT covariance networks in MS are characterized by increased segregation with an enhancement of local properties, as well as a shift towards more regular networks in advanced disease^22–25^. Convergent findings have emerged from studies of DTI-based structural networks^26,27^ and MEG-based functional connectivity in MS patients^24^. Together, the evidence points to a pathological shift towards more segregated and regular networks in MS, and our findings suggest that the MS patients who experience these shifts may also lose their capacity to experience placebo effects. It follows that placebo responses may require a cortical network topology that favours distributed processing and functional integration.

One interpretation of our findings is that in placebo non-responders, the disease process may have resulted in more synchronized cortical tissue loss across different brain regions leading to increasingly correlated cortical thickness values. This interpretation is supported by the associations we found between lesion load and regional CT in placebo non-responders only. Although white matter demyelination is thought to drive neuronal degeneration in MS, there is evidence that the two can occur independently, and laminar contributions to cortical neuronal loss (and hence thinning) may differ depending on whether it is related to versus independent from white matter demyelination^48^. In the non-responders, lesion load significantly predicted cortical thinning in a substantial portion of the right hemisphere, including primary sensory and motor areas and somatosensory, visual and auditory association areas. Together with precuneus and insula, association areas have been identified as hubs by graph theoretical studies of structural connectivity^49^. Insults to these hubs and their connections are likely to result in changes in network organization with major implications for functional integration of neural activity^50,51^. Synchronous loss of neurons in these regions and of projections between them could impair associative processes enabling placebo responses, such as integrating interoceptive appraisals with expectancy of therapeutic benefit. Precuneus and insula, which are key structures for self-referential thinking and interoceptive awareness, may play a central role in such expectancy-informed appraisals. Loss of projections between these regions and shared deep nuclei could also result in an organization increasingly dependent on local connections.

Although the causes of the differential associations between CT and lesion load in placebo responders versus non-responders remain undetermined, lesion characteristics may play a role. Cortical grey matter loss in MS may arise from a combination of primary pathological processes and secondary effects of white matter damage. Regarding the latter, chronic inactive lesions are more likely to be associated with axonal degeneration^52^. Although both placebo responders and non-responders had relatively advanced disease (10–20 years, median EDSS of 4.0), at which point most lesions are inactive^53^, and gadolinium enhancing lesions were observed in a minority of patients, responders had a significantly higher incidence of active lesions. The same subset of patients also had more lesions that became enlarged at 24 or 48 weeks relative to baseline, suggesting either expanding inflammatory activity or slowly expanding “smoldering” lesions^53^. Based on this, and given equivalent lesion load in the two groups, it is plausible that placebo non-responders had a higher proportion of inactive lesions, more likely to be chronic and to reflect axonal loss potentially driving synchronized loss of cortical tissue. In addition, the absence of active inflammation in non-enhanced lesions may have caused their volume to be reduced compared to that of active lesions, resulting in an apparently smaller lesion load. Lesion location, on the other hand, did not drive differential associations between lesion load and CT, as lesion maps did not differ between responders and non-responders.

Whether the network characteristics we observed to be associated with the absence of placebo response would generalize to other types of placebo responses or other patient groups remains to be determined. This could be tested by applying graph theoretical analysis in future studies of the neural mechanisms of placebo responses or to existing published datasets. Thus far, graph theory has seldom been used to study neural correlates of placebo responses. We are aware of only one study using graph theory metrics of DTI-based structural networks to predict placebo response in migraine patients^4^. In that study, increased global and local efficiency at baseline inversely predicted placebo analgesic response to sham acupuncture, which is broadly consistent with our findings.

Our study had several limitations. First, to maximize sample size, sham and venoplasty participants were combined under the reasonable assumption that both interventions were effectively sham, considering that venoplasty for MS was found ineffective in two independent trials. Indeed, the responder group included non-significantly more sham participants (Table 3). Moreover, supplementary analyses performed separately in sham and venoplasty groups yielded similar trends (Supplement, Tables S5–S6), highlighting the robustness of the differences in CT networks between responders and non-responders. We consider the findings from the full sample more likely to be reliable. Second, we excluded some patients due to poor MRI quality (see “Methods” section). Third, responders and non-responders were identified based on subjective self-report of health-related quality of life. Hence, our findings may not generalize to placebo responses manifesting as more objective clinical improvement, which we did not observe in the trial^18^. However, even subjective placebo responses can be quite compelling for the patients: in the case of venoplasty, they may have contributed to fueling the efforts of patient advocacy groups to legitimize the procedure in the face of skepticism from the scientific community^54^. Fourth, in a trial with a 48-week follow-up, placebo response is necessarily confounded with the natural course of the disease including relapses, remissions and regression to the mean. We consider substantial effects of such confounds unlikely because (1) we adjusted our measure of placebo response for baseline scores to minimize the impact of regression to the mean; and (2) the incidence of active lesions did not change in either group over the course of the trial, making it unlikely that placebo response was driven by remissions. The latter is further supported by the absence of placebo response on more objective clinician-rated measures, such as the Expanded Disability Status Scale (EDSS). Fifth, the non-responder group had non-significantly poorer clinical scores on some measures, raising the possibility of our brain morphological findings simply reflecting the neural correlates of disease severity rather than placebo response. Although possible, we consider this unlikely, since the findings remained unchanged in the models including baseline EDSS scores (see “Methods” section). Finally, our graph theoretical analysis was based on cortical thickness covariance patterns, which do not represent either true structural connectivity or a direct measure of functional connectivity. Rather, they are thought of as an indirect reflection of functional connectivity between brain regions. Resting state fMRI data could have provided valuable direct information regarding functional connectivity differences between placebo responders and non-responders. Unfortunately, no resting state sequences were collected as part of this trial. Although DTI sequences were available, DTI as measure of structural connectivity is problematic in advanced MS, as white matter lesions present a challenge for tract identification.

In conclusion, our findings demonstrate that the absence of placebo response in MS is associated with (1) a more regular and segregated CT topology, (2) cortical tissue loss related to white matter pathology, and (3) lower lesion activity. Considering that placebo response is a constituent of active therapeutic response, these morphometric characteristics may by extension predict responses to active therapies. Finally, our findings highlight graph theory as a promising tool for future studies of the neurobiology of placebo responses.

## Availability of data and materials

Data and analysis code are available upon request.

## Supplementary Information

Supplementary Information.
