# Supplementary material for: Cortical morphology predicts placebo response in multiple sclerosis
Source: Sci Rep. 2022 Jan 14;12:732. doi: 10.1038/s41598-021-04462-7 (PMC8760243; doi:10.1038/s41598-021-04462-7)
Supplement: Supplementary file 1 — Supplementary Information. [file 41598_2021_4462_MOESM1_ESM.docx]

**Supplementary Methods**

**Participants**

Participants with relapsing remitting (RRMS), secondary (SPMS) and primary progressive (PPMS) MS were recruited between May 29, 2013 and Aug 19, 2015 from four Canadian academic centers: 1) University of British Columbia Hospital, Vancouver; 2) Health Sciences Centre, Winnipeg; 3) CHUM, Hôpital Notre-Dame, Montreal; 4) Hôpital Enfant-Jesus, Québec. Inclusion criteria were: age 18-65 years, diagnosis of definite MS by the 2010 McDonald criteria ^1^, an Expanded Disability Status Score (EDSS) ^2^ between 0 (i.e. minimal disability) and 6.5 (i.e. using bilateral aids to walk), neurologically stable disease within the 30 days prior to screening, and fulfillment of at least two ultrasound criteria for CCSVI see ^3^ for a detailed description of the trial’s methods and entry criteria. Participants on standard disease-modifying therapies were permitted to continue on the medication, and changes were allowed for on study relapses after randomization. Exclusion criteria were treatment with vasodilators, parasympathomimetics, sympathicolytics, calcium channel blockers, previous venoplasty and/or stenting, previous jugular or subclavian central line or major neck surgery or radiation, previous contrast allergy, inability to undergo MRI, and inadequate medical records confirming diagnosis and disease course. The clinical research ethics boards at the four participating centers approved the study protocol, and participants gave written informed consent.

**Trial Procedure**

Eligible participants were randomized 1:1 to either sham or active balloon venoplasty of all narrowed veins under study. Stratified randomization (RRMS versus progressive MS course) at each site was completed by a permuted-block size of six. Venography was performed under conscious sedation and the duration of time within the angiography suite was uniform for both venoplasty and sham treated participants. A 5-French diagnostic catheter was introduced through the common femoral vein to selectively catheterize the right and left internal jugular veins as well as the azygos vein. The venoplasty participants were treated with an angioplasty balloon 2mm greater than the nominal vein diameter which was inflated for 60 seconds. The participants randomized to sham had a catheter that was advanced across the stenosis and left for 60 seconds.

**Supplementary Analyses**

***CIVET pipeline***

The CIVET pipeline included the following steps. 1) The native MRIs were registered to standard space using a 9-parameter nonlinear transformation ^4^. For the controls, the MNI-152 template was used, whereas for the MS patients, a high-resolution Alzheimer’s disease template provided by CIVET was used. Because of paraventricular brain atrophy in the MS patients, the AD template produced superior registration, and since our hypotheses pertained to the MS groups, we chose to use the template that was the most optimal for the patients. Simultaneous non-uniformity correction was performed using N3 algorithms ^5^. 3) The registered corrected images were classified into white matter, gray matter, CSF, and background, using an artificial neural network classifier ^6^. 4) Binary volumes consisting of gray matter voxels and white matter voxels were then extracted from classified images, smoothed using an 8-mm FWHM smoothing kernel and used in the subsequent voxel-based morphometry analyses (see below). 5) Cortical surfaces were extracted from the classified images using the Constrained Laplacian Anatomic Segmentation Using Proximities (CLASP) surface extraction procedure ^7^ ^8^. This process generates a triangulated mesh at the interface of gray matter and white matter and expands the mesh outwards toward the pial surface. 6) Cortical thickness was measured in native space as the distance between corresponding vertices on the inner and outer surfaces of the mesh across 40,962 vertices in each hemisphere ^9^. The cortical thickness maps were then blurred using a 20 mm surface based kernel ^10^. Quality control was performed using a combination of the CIVET QC tool and visual inspection of the outputs.

***Voxel-based morphometry***

Voxel-based morphometry was conducted using the PET and VBM module of Statistical Parametric Mapping (SPM 12, RRID:SCR_007037). The grey and white matter volumes were compared using voxel-wise two sample t-tests with our standard covariates plus total brain volume. Dimensional associations between volumes and the degree of placebo response, represented by the AUC change in MSQOL-54 scores adjusted for baseline scores, were also examined using multiple regression models with the same covariates. The resulting statistical maps were thresholded using family-wise error corrected threshold of p = 0.05.

***Lesion location analysis***

The analysis of lesion location was performed in FSL (Functional MRI of the Brain Software Library, http://[www.fmrib.ox.ac.uk/fsl](http://www.fmrib.ox.ac.uk/fsl), RRID:SCR_002823). Only the lesions identified on FLAIR scans were considered for this analysis, as there were too few participants with gadolinium enhanced lesions on the baseline scans (four non-responders; six responders, Table 3). FLAIR lesions masks were moved into the MNI-152 space, and lesion probability maps were created for the placebo responder and for the placebo non-responder groups by averaging the registered binary masks across patients in each group using the *fslmaths* utility. These voxel-wise maps representing the probability of each voxel being lesional were compared statistically between the two groups using the *Randomise* algorithm ^11^, which uses non-parametric permutation inference to threshold a voxel-wise statistical map produced, in this case, by voxel-wise unpaired t-tests on the two groups.

**Supplementary Results**

Placebo responders and non-responders did not differ in terms of normalized brain volumes at baseline or percent brain volume change over the 48 weeks of follow-up, although there was progressive atrophy in both groups (b = -0.31, SE = 0.09, t = -3.45, p = 0.0009). Voxel-based morphometry did not reveal any significant group differences in regional grey or white matter volume. There were also no significant group differences in white matter lesion load (see Table S1 for details).

Table S1: Brain morphometric characteristics

| **Measure (SD)** | **Non-responder (n = 45)** | **Responder (n = 43)** | **p** |
| --- | --- | --- | --- |
| Normalized brain parenchymal fraction (baseline) | 0.75 (0.03) | 0.77 (0.03) | 0.39 |
| % Change in brain volume from baseline |  |  |  |
| 24 weeks | -0.40 (0.50) | -0.43 (0.38) | 0.78 |
| 48 weeks | -0.70 (0.60) | -0.61 (0.61) | 0.65 |
| WM lesion load (mm^3^) |  |  |  |
| Baseline | 12031.63 (12002.73) | 12269.53 (14538.12) | 0.78 |
| 24 weeks | 12681.97 (12185.17) | 12581.44 (14527.14) | 0.92 |
| 48 weeks | 12792.65 (11875.89) | 12907.21 (14698.09) | 0.87 |

Table S2. Permutation test p-values for placebo responders versus non-responders at different sparsity thresholds.

| **Sparsity**  **threshold** | **Mean**  **clustering**  **coefficient** | **Normalized**  **clustering**  **coefficient** | **Characteristic**  **pathlength** | **Normalized**  **pathlength** | **Small world index** |
| --- | --- | --- | --- | --- | --- |
| 0.10 | **0.00*** | **0.02*** | 0.06† | 0.06† | **0.01*** |
| 0.11 | **0.01*** | **0.03*** | **0.021*** | **0.02*** | **0.00*** |
| 0.13 | **0.02*** | **0.02*** | 0.51 | 0.50 | 0.21 |
| 0.14 | **0.04*** | **0.03*** | 0.54 | 0.53 | 0.20 |
| 0.16 | **0.04*** | 0.06† | 0.52 | 0.52 | 0.14 |
| 0.17 | 0.07† | **0.04*** | 0.46 | 0.46 | 0.07† |
| 0.18 | 0.07† | 0.06† | 0.39 | 0.39 | **0.04*** |
| 0.20 | 0.13 | 0.19 | 0.40 | 0.40 | 0.09† |
| 0.21 | **0.05*** | 0.07† | 0.40 | 0.40 | **0.03*** |
| 0.22 | **0.03*** | **0.04*** | 0.31 | 0.31 | **0.01*** |
| 0.24 | **0.03*** | **0.03*** | 0.25 | 0.24 | **0.01*** |
| 0.25 | **0.04*** | **0.03*** | 0.22 | 0.22 | **0.01*** |
| 0.27 | **0.04*** | **0.04*** | 0.21 | 0.21 | **0.01*** |
| 0.28 | **0.02*** | **0.02*** | 0.19 | 0.19 | **<0.001*** |
| 0.29 | **0.02*** | **0.02*** | 0.16 | 0.16 | **<0.001*** |
| 0.31 | **0.02*** | **0.03*** | 0.15 | 0.15 | **<0.001*** |
| 0.32 | **0.02*** | **0.02*** | 0.12 | 0.12 | **<0.001*** |
| 0.33 | **0.02*** | **0.02*** | 0.11 | 0.11 | **0.01*** |
| 0.35 | **0.03*** | **0.02*** | 0.10 | 0.10 | **0.01*** |
| 0.36 | **0.02*** | **0.02*** | 0.09† | 0.09† | **0.01*** |
| 0.38 | **0.02*** | **0.02*** | 0.08† | 0.08† | **0.01*** |
| 0.39 | **0.02*** | **0.02*** | 0.07† | 0.07† | **0.01*** |
| 0.40 | **0.02*** | **0.02*** | 0.07† | 0.07† | **0.01*** |
| 0.42 | **0.02*** | **0.02*** | 0.06† | 0.06† | **0.01*** |
| 0.43 | **0.02*** | **0.02*** | 0.06† | 0.06† | **0.01*** |
| 0.44 | **0.02*** | **0.02*** | 0.05† | 0.05† | **0.01*** |
| 0.46 | **0.02*** | **0.03*** | 0.05† | 0.05† | **0.03*** |
| 0.47 | **0.05*** | **0.04*** | 0.05† | 0.05† | **0.03*** |
| 0.49 | **0.05*** | **0.05*** | 0.05† | 0.05† | **0.04*** |
| 0.50 | **0.03*** | **0.03*** | 0.05† | 0.06† | **0.03*** |

*p ≤ 0.05; †p ≤ 0.1

Table S3. Permutation test p-values for controls versus placebo non-responders at different sparsity thresholds.

| **Sparsity**  **threshold** | **Mean**  **clustering**  **coefficient** | **Normalized**  **clustering**  **coefficient** | **Characteristic**  **pathlength** | **Normalized**  **pathlength** | **Small world index** |
| --- | --- | --- | --- | --- | --- |
| 0.10 | 0.09† | **0.05*** | 0.06† | 0.08† | **0.03*** |
| 0.11 | 0.06† | **0.05*** | 0.49 | 0.06† | **0.01*** |
| 0.13 | 0.07† | 0.23 | 0.50 | 0.49 | 0.41 |
| 0.14 | 0.08† | 0.14 | 0.49 | 0.50 | 0.34 |
| 0.16 | 0.15 | 0.15 | 0.50 | 0.49 | 0.28 |
| 0.17 | 0.11 | 0.12 | 0.40 | 0.50 | 0.23 |
| 0.18 | 0.11 | 0.09† | 0.42 | 0.40 | 0.10 |
| 0.20 | 0.11 | 0.14 | 0.40 | 0.42 | 0.09† |
| 0.21 | **0.04*** | 0.07† | 0.35 | 0.40 | **0.04*** |
| 0.22 | **0.05*** | **0.03*** | 0.32 | 0.35 | **0.02*** |
| 0.24 | **0.05*** | 0.05† | 0.31 | 0.32 | **0.02*** |
| 0.25 | **0.05*** | 0.05† | 0.25 | 0.31 | **0.02*** |
| 0.27 | **0.04*** | **0.03*** | 0.20 | 0.26 | **0.01*** |
| 0.28 | **0.04*** | **0.05*** | 0.18 | 0.20 | **0.02*** |
| 0.29 | 0.06† | 0.08† | 0.17 | 0.18 | **0.02*** |
| 0.31 | 0.06† | 0.05† | 0.14 | 0.17 | **0.02*** |
| 0.32 | **0.04*** | **0.05*** | 0.11 | 0.14 | **0.02*** |
| 0.33 | **0.05*** | **0.04*** | 0.11 | 0.11 | **0.02*** |
| 0.35 | 0.06† | 0.06† | 0.08† | 0.11 | **0.03*** |
| 0.36 | **0.04*** | **0.05*** | 0.06† | 0.08† | **0.02*** |
| 0.38 | **0.04*** | **0.03*** | **0.05*** | 0.06† | **0.02*** |
| 0.39 | **0.02*** | **0.03*** | **0.05*** | 0.05† | **0.02*** |
| 0.40 | **0.02*** | **0.02*** | **0.05*** | **0.05*** | **0.02*** |
| 0.42 | **0.01*** | **0.01*** | **0.04*** | **0.05*** | **0.01*** |
| 0.43 | **0.01*** | **0.01*** | **0.03*** | **0.04*** | **0.01*** |
| 0.44 | **0.01*** | **0.01*** | **0.03*** | **0.03*** | **0.01*** |
| 0.46 | **0.01*** | **0.01*** | **0.03*** | **0.03*** | **0.01*** |
| 0.47 | **0.02*** | **0.02*** | **0.03*** | **0.03*** | **0.02*** |
| 0.49 | **0.02*** | **0.02*** | **0.03*** | **0.03*** | **0.02*** |
| 0.50 | **0.02*** | **0.02*** | 0.07† | **0.03*** | **0.02*** |

*p ≤ 0.05; †p ≤ 0.1

Table S4. Permutation test p-values for controls versus placebo responders at different sparsity thresholds.

| **Sparsity**  **threshold** | **Mean**  **clustering**  **coefficient** | **Normalized**  **clustering**  **coefficient** | **Characteristic**  **pathlength** | **Normalized**  **pathlength** | **Small world index** |
| --- | --- | --- | --- | --- | --- |
| 0.10 | 0.83 | 0.50 | 0.26 | 0.27 | 0.35 |
| 0.11 | 0.66 | 0.54 | 0.48 | 0.49 | 0.51 |
| 0.13 | 0.67 | 0.63 | 0.41 | 0.43 | 0.55 |
| 0.14 | 0.57 | 0.59 | 0.44 | 0.42 | 0.53 |
| 0.16 | 0.71 | 0.73 | 0.43 | 0.43 | 0.65 |
| 0.17 | 0.59 | 0.69 | 0.58 | 0.57 | 0.71 |
| 0.18 | 0.60 | 0.62 | 0.38 | 0.39 | 0.52 |
| 0.20 | 0.39 | 0.43 | 0.38 | 0.37 | 0.36 |
| 0.21 | 0.39 | 0.42 | 0.38 | 0.38 | 0.37 |
| 0.22 | 0.51 | 0.53 | 0.42 | 0.42 | 0.48 |
| 0.24 | 0.45 | 0.54 | 0.47 | 0.47 | 0.51 |
| 0.25 | 0.41 | 0.38 | 0.52 | 0.53 | 0.42 |
| 0.27 | 0.40 | 0.40 | 0.45 | 0.45 | 0.40 |
| 0.28 | 0.59 | 0.56 | 0.43 | 0.43 | 0.50 |
| 0.29 | 0.65 | 0.61 | 0.43 | 0.43 | 0.55 |
| 0.31 | 0.62 | 0.55 | 0.42 | 0.42 | 0.49 |
| 0.32 | 0.55 | 0.51 | 0.45 | 0.45 | 0.48 |
| 0.33 | 0.43 | 0.48 | 0.43 | 0.43 | 0.45 |
| 0.35 | 0.47 | 0.45 | 0.43 | 0.43 | 0.44 |
| 0.36 | 0.46 | 0.46 | 0.40 | 0.40 | 0.42 |
| 0.38 | 0.42 | 0.42 | 0.39 | 0.39 | 0.40 |
| 0.39 | 0.34 | 0.33 | 0.41 | 0.41 | 0.36 |
| 0.40 | 0.33 | 0.33 | 0.40 | 0.40 | 0.35 |
| 0.42 | 0.30 | 0.31 | 0.40 | 0.40 | 0.34 |
| 0.43 | 0.30 | 0.33 | 0.39 | 0.39 | 0.35 |
| 0.44 | 0.30 | 0.33 | 0.38 | 0.38 | 0.34 |
| 0.46 | 0.29 | 0.29 | 0.37 | 0.37 | 0.32 |
| 0.47 | 0.28 | 0.28 | 0.35 | 0.35 | 0.31 |
| 0.49 | 0.27 | 0.26 | 0.35 | 0.35 | 0.30 |
| 0.50 | 0.31 | 0.32 | 0.34 | 0.34 | 0.33 |

*p ≤ 0.05; †p ≤ 0.1

Table S5. Permutation test p-values for placebo responders versus non-responders including only sham-treated participants at different sparsity thresholds.

| **Sparsity**  **threshold** | **Mean**  **clustering**  **coefficient** | **Normalized**  **clustering**  **coefficient** | **Characteristic**  **pathlength** | **Normalized**  **pathlength** | **Small world index** |
| --- | --- | --- | --- | --- | --- |
| 0.10 | **0.01*** | **0.02*** | 0.82 | 0.83 | 0.33 |
| 0.11 | **0.01*** | **0.01*** | 0.84 | 0.85 | 0.27 |
| 0.13 | **<0.00*** | **<0.00*** | 0.83 | 0.83 | 0.12 |
| 0.14 | **<0.00*** | **0.01*** | 0.86 | 0.86 | 0.16 |
| 0.16 | **0.01*** | **0.02*** | 0.93 | 0.93 | 0.35 |
| 0.17 | **<0.001*** | **<0.001*** | 0.91 | 0.91 | 0.16 |
| 0.18 | **<0.001*** | **<0.001*** | 0.79 | 0.79 | **0.05*** |
| 0.20 | **<0.001*** | **<0.001*** | 0.73 | 0.73 | **0.03*** |
| 0.21 | **<0.001*** | **<0.001*** | 0.74 | 0.74 | **0.03*** |
| 0.22 | **0.03*** | **0.04*** | 0.68 | 0.68 | 0.10 |
| 0.24 | **0.04*** | **0.04*** | 0.65 | 0.65 | 0.08† |
| 0.25 | **0.04*** | **0.04*** | 0.59 | 0.58 | 0.07† |
| 0.27 | 0.06† | **0.10*** | 0.59 | 0.59 | 0.11 |
| 0.28 | 0.06† | 0.08† | 0.55 | 0.55 | 0.10 |
| 0.29 | 0.06† | **0.05*** | 0.45 | 0.45 | 0.06† |
| 0.31 | 0.08† | 0.07† | 0.43 | 0.43 | 0.08† |
| 0.32 | 0.07† | 0.07† | 0.39 | 0.39 | 0.08† |
| 0.33 | **0.05*** | 0.06† | 0.42 | 0.42 | 0.07† |
| 0.35 | **0.05*** | **0.05*** | 0.36 | 0.36 | 0.07† |
| 0.36 | **0.03*** | **0.03*** | 0.27 | 0.27 | **0.04*** |
| 0.38 | **0.04*** | **0.04*** | 0.26 | 0.26 | **0.04*** |
| 0.39 | **0.04*** | **0.04*** | 0.25 | 0.25 | 0.06† |
| 0.40 | **0.04*** | **0.04*** | 0.25 | 0.25 | 0.06† |
| 0.42 | **0.05*** | **0.05*** | 0.23 | 0.23 | 0.07† |
| 0.43 | 0.06† | 0.06† | 0.24 | 0.24 | 0.08† |
| 0.44 | 0.06† | 0.07† | 0.24 | 0.24 | 0.09† |
| 0.46 | 0.07† | 0.07† | 0.23 | 0.23 | 0.09† |
| 0.47 | 0.08† | 0.09† | 0.22 | 0.22 | 0.11 |
| 0.49 | 0.11 | 0.11 | 0.22 | 0.22 | 0.13 |
| 0.50 | 0.13 | 0.12 | 0.22 | 0.22 | 0.15 |

*p ≤ 0.05; †p ≤ 0.1; sham responders: n = 25; sham non-responders: n = 21

Table S6: Permutation test p-values for placebo responders versus non-responders including only venoplasty-treated participants at different sparsity thresholds.

| **Sparsity**  **threshold** | **Mean**  **clustering**  **coefficient** | **Normalized**  **clustering**  **coefficient** | **Characteristic**  **pathlength** | **Normalized**  **pathlength** | **Small world index** |
| --- | --- | --- | --- | --- | --- |
| 0.10 | 0.28 | 0.31 | **<0.001*** | **<0.001*** | **<0.001*** |
| 0.11 | 0.40 | 0.49 | 0.07† | 0.08† | 0.07† |
| 0.13 | 0.33 | 0.20 | **0.03*** | **0.03*** | **0.02*** |
| 0.14 | 0.32 | 0.34 | **0.04*** | **0.04*** | **0.04*** |
| 0.16 | 0.53 | 0.50 | **0.01*** | **0.01*** | 0.06† |
| 0.17 | 0.42 | 0.43 | **0.02*** | **0.02*** | 0.06† |
| 0.18 | 0.33 | 0.32 | 0.10† | 0.10† | 0.09† |
| 0.20 | 0.30 | 0.17 | 0.17 | 0.17 | 0.09† |
| 0.21 | 0.29 | 0.21 | 0.19 | 0.19 | 0.12 |
| 0.22 | 0.26 | 0.34 | 0.18 | 0.18 | 0.17 |
| 0.24 | 0.33 | 0.34 | 0.08† | 0.08† | 0.13 |
| 0.25 | 0.22 | 0.21 | 0.13 | 0.13 | 0.10 |
| 0.27 | 0.19 | 0.22 | 0.10† | 0.10† | 0.11 |
| 0.28 | 0.16 | 0.16 | 0.09† | 0.09† | 0.08† |
| 0.29 | 0.13 | 0.13 | 0.09† | 0.09† | 0.07† |
| 0.31 | 0.13 | 0.12 | 0.07† | 0.07† | 0.06† |
| 0.32 | 0.12 | 0.15 | 0.06† | 0.06† | 0.08† |
| 0.33 | 0.12 | 0.10† | 0.06† | 0.06† | 0.06† |
| 0.35 | 0.11 | 0.12 | 0.06† | 0.06† | 0.07† |
| 0.36 | 0.11 | 0.10† | **0.05*** | **0.05*** | 0.06† |
| 0.38 | 0.11 | 0.10† | **0.04*** | **0.04*** | 0.06† |
| 0.39 | 0.10† | 0.08† | **0.04*** | **0.04*** | 0.05† |
| 0.40 | 0.06† | 0.09† | **0.05*** | **0.05*** | 0.05† |
| 0.42 | 0.06† | 0.06† | **0.05*** | **0.05*** | **0.05*** |
| 0.43 | 0.07† | 0.06† | **0.05*** | **0.05*** | **0.05*** |
| 0.44 | 0.06† | 0.06† | **0.05*** | **0.05*** | **0.05*** |
| 0.46 | 0.06† | 0.06† | **0.04*** | **0.05*** | **0.04*** |
| 0.47 | 0.07† | 0.07† | **0.04*** | **0.04*** | **0.05*** |
| 0.49 | 0.07† | 0.07† | **0.04*** | **0.04*** | **0.05*** |
| 0.50 | 0.06† | 0.06† | **0.04*** | **0.04*** | **0.04*** |

*p ≤ 0.05; †p ≤ 0.1; venoplasty responders: n = 18; venoplasty non-responders: n = 24

**References**

1. Polman, C. H. *et al.* Diagnostic Criteria for Multiple Sclerosis : 2010 Revisions to the McDonald Criteria. *Ann. Neurol.* **69**, 292–302 (2011).

2. Kurtzke, J. F. Rating neurologic impairment in multiple sclerosis : An expanded disability status scale ( EDSS ). *Neurology* **33**, 1444–1453 (1983).

3. Traboulsee, A. L. *et al.* Safety and e fficacy of venoplasty in MS: a randomized, double-blind, sham-controlled phase II trial. *Neurology* **91**, e1660–e1668 (2018).

4. Collins, L., Neelin, P., Peters, T. M. & Evans, A. C. Automatic 3D intersubject registration of MR volumetric data in standardized talairach space. *J. Comput. Assist. Tomogr.* **18**, 192–205 (1994).

5. Sled, J. G., Zijdenbos, A. P. & Evans, A. C. A Nonparametric Method for Automatic Correction of Intensity Nonuniformity in MRI Data. *IEEE Trans. Med. Imaging* **17**, 87–97 (1998).

6. Zijdenbos, A. P., Forghani, R. & Evans, A. C. Automatic “ Pipeline ” Analysis of 3-D MRI Data for Clinical Trials : Application to Multiple Sclerosis. *IEEE Trans. Med. Imaging* **21**, 1280–1291 (2002).

7. Kim, J. S. *et al.* Automated 3-D extraction and evaluation of the inner and outer cortical surfaces using a Laplacian map and partial volume effect classification. *Neuroimage* **27**, 210–221 (2005).

8. Macdonald, D., Kabani, N., Avis, D. & Evans, A. C. Automated 3-D Extraction of Inner and Outer Surfaces of Cerebral Cortex from MRI. *Neuroimage* **356**, 340–356 (2000).

9. Lerch, J. P. & Evans, A. C. Cortical thickness analysis examined through power analysis and a population simulation. *Neuroimage* **24**, 163–173 (2005).

10. Chung, M. K. *et al.* Deformation-based surface morphometry applied to gray matter deformation. *Neuroimage* **18**, 198–213 (2003).

11. Winkler, A. M., Ridgway, G. R., Webster, M. A., Smith, S. M. & Nichols, T. E. Permutation inference for the general linear model. *Neuroimage* **92**, 381–397 (2014).
